# αKlotho decreases after reduced weight-bearing from both spaceflight and hindlimb unloading

**DOI:** 10.1038/s41526-022-00203-w

**Published:** 2022-06-02

**Authors:** Jeffrey S. Willey, Serena Aunon-Chancellor, Lauren A. Miles, Joseph E. Moore, Xiao W. Mao, Robert W. Wallace, Matthew C. Foy

**Affiliations:** 1 Department of Radiation Oncology, Wake Forest School of Medicine, Section of Radiation Biology, Winston-Salem, NC USA; 2 Department of Internal Medicine, Louisiana State University Medical Center, Baton Rouge, LA USA; 3 Department of Nephrology, Wake Forest School of Medicine, Winston-Salem, NC USA; 4 Department of Basic Sciences, Loma Linda University, Loma Linda, CA USA; 5 Department of Internal Medicine, Louisiana State University Medical Center, Division of Nephrology, Baton Rouge, LA USA

**Keywords:** Predictive markers, Physiology

## Abstract

Alpha(α)Klotho, a soluble transmembrane protein, facilitates calcium-phosphorus homeostasis through feedback between bone and kidney and is a potential systemic biomarker for bone-kidney health during spaceflight. We determined if: (1) plasma αKlotho was reduced after both spaceflight aboard the ISS and hindlimb unloading (HU); and (2) deficiency could be reversed with exercise. Both spaceflight and HU lowered circulating plasma αKlotho: plasma αKlotho recovered with exercise after HU.

## Introduction

Atrophy of skeletal elements has long been identified as a hazard for the success of long-duration missions and astronaut quality of life^1^. US astronauts aboard the International Space Station (ISS) exhibit loss of bone density when comparing pre- and post-flight DXA scan measurements^2,3^. Bone density reduction for a Mars mission has been estimated of up to 36%^4^. Recovery from bone degradation appears to be incomplete^5,6^.

The role of the kidneys in skeletal homeostasis is complex. Recent research interest includes the interplay between αKlotho and Fibroblast Growth Factor 23 (FGF23)^7^. Alpha Klotho is secreted predominantly in the kidneys, whereas FGF23 is secreted by osteocytes^8^. FGF23 binds to αKlotho, and increases urine phosphorous excretion^9^. Both are required, as seen in mice that demonstrate phosphate retention if either component is deficient^10^. FGF23 also increases calcium reabsorption^7^. Through these mechanisms, FGF23 eliminates excess phosphorus and increases plasma calcium, with subsequent decreased calcitriol synthesis and parathyroid hormone (PTH) release. In non-weight-bearing climates with known bone density loss, αKlotho and FGF23 may play a role in skeletal homeostasis.

The current spaceflight countermeasures aimed at preventing bone loss provide incomplete protection^6,11^. The Advanced Resistive Exercise Device (ARED) is utilized for exercise aboard the ISS^12^. Use of the ARED as a single countermeasure largely maintains preflight skeletal health over a 6-month mission in orbit^11^, but bone loss is observed by 1 year post-flight, indicating progressive atrophy despite a return to full weight-bearing and from astronauts also receiving bisphosphonate therapy^11^. Additionally, a device as large and complex as ARED is likely not feasible on an exploration class mission due to limitations in mass and habitable space. These findings add concerns for the current ability to preserve bone health outside low earth orbit.

Currently, no reliable real-time analysis of biomarkers or skeletal imaging on orbit exists. Identification of novel circulating biomarkers for musculoskeletal health, such as αKlotho, could prove crucial to protective measures as the space community turns its eyes towards Mars.

This pilot investigation measured circulating αKlotho levels in mice after exposure to two reduced weight-bearing conditions: microgravity aboard the ISS, and after hind limb unloading (HU)^13^. Importantly, musculoskeletal degradation has previously been described in hindlimbs from these same mice after 35 days in microgravity and HU^14,15^, observing recovery of joint health upon return to full weight-bearing with exercise^15^. Likewise, this study examined if deficits in circulating αKlotho after periods HU could be recovered after return to full-weight bearing, with/without performing exercise. Changes in α-Klotho after acute bouts of exercise are inconsistent when examining resistance vs aerobic exercise (ref. ^16^), with long term exercise exhibiting elevated αKlotho in sedentary individuals (ref. ^17^). This hypothesis-generating research aims to assess if αKlotho can serve as a circulating biomarker for bone status, both in-flight and upon return to weight-bearing.

**Fig. 1:**
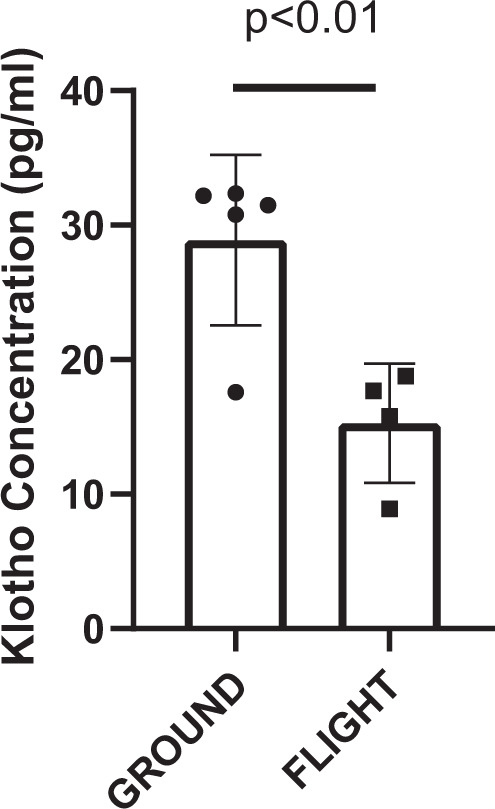
αKlotho concentration is reduced after spaceflight. FLIGHT mice exhibited lower αKlotho vs GROUND. *P* value from unpaired t test provided above bars. Bars: Mean(SD).

**Fig. 2:**
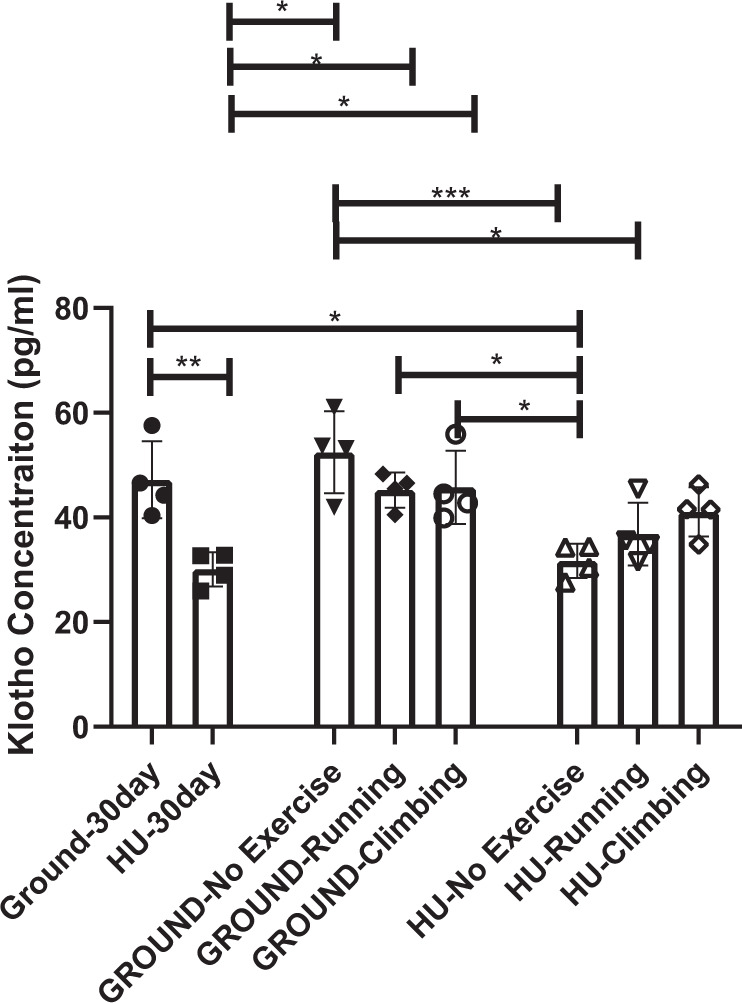
αKlotho concentration is reduced after hind limb unloading, but can recover with subsequent exercise. αKlotho was lower than GROUND in plasma after 30 days HU, but recovery occurred with exercise *p ≤ 0.05; **p < 0.01; ***p < 0.001 determined via Tukey’s Post Hoc test after performed after ANOVA. Bars: Mean(SD).

**Fig. 3:**
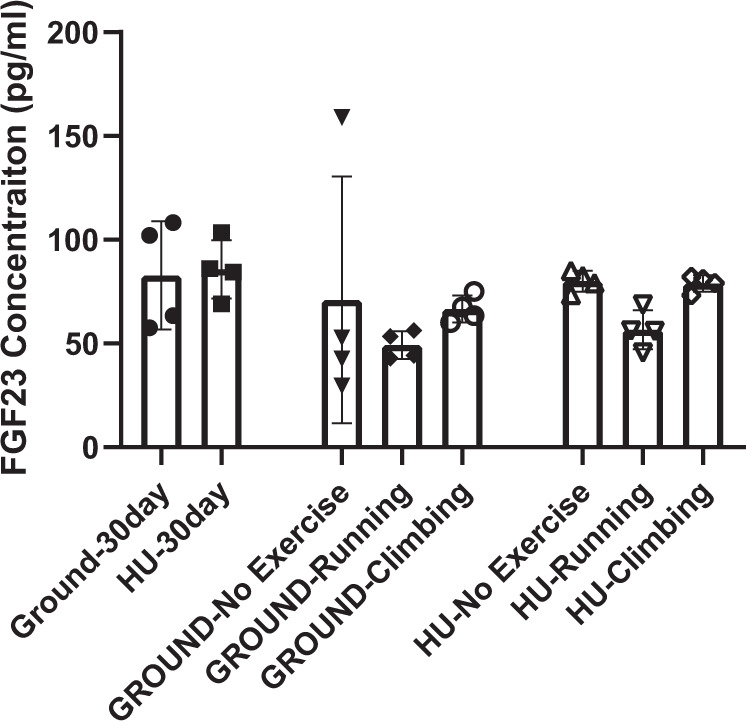
Plasma FGF23 was unaltered after hind limb unloading. Plasma FGF23 was similar across groups and time points. Bars: Mean(SD).

Data are presented mean(SD). Plasma αKlotho in pg/ml was ~50% lower (Fig. 1) after 35 days in orbit aboard the ISS from the FLIGHT mice vs GROUND. No differences were observed between groups for FGF23 or inorganic phosphorus (Pi) concentration (Table 1). Additionally, gastrocnemius muscle mass (g) was lower (*p* = 0.0035) in FLIGHT vs GROUND after time in orbit aboard the ISS (Table 1). For the hind limb unloading study, plasma αKlotho after 30 days of tail suspension [HU-30day] was 36% lower vs plasma αKlotho from mice that remained full weight bearing [GROUND-30day; *p* < 0.01; Fig. 2]. Likewise, αKlotho was lower in mice after the initial 30 days of HU vs all mice that were full-weight bearing throughout the entire 80 day study, with or without exercise [GROUND-No exercise; GROUND-Climbing; and GROUND-Running; *p* < 0.05 for all comparisons)]. In contrast, after returning to full weight-bearing from the 30 day period of HU, plasma αKlotho levels remained lower in the mice that performed no exercise [HU-No Exercise] vs plasma from the full weight bearing mice as measured on Day 30 (*p* < 0.05). Additionally, these mice that had not performed exercise after the 30 day period of HU were also significantly lower than all GROUND groups on Day 80, regardless of exercise (GROUND-No exercise; GROUND-Climbing; GROUND-Running). In contrast, running exercise for 49 days after the initial 30 day HU period [HU-Running] resulted in partial recovery of plasma Klotho, with no difference vs the full weight bearing group at Day 30 (GROUND-30 Day), but still having lower concentration (*p* < 0.05) vs GROUND-No Exercise. Climbing exercise [HU-Climbing] for 49 days provided full recovery of αKlotho (Fig. 2), being similar to all GROUND groups. No differences were observed between any groups for FGF23 concentration (Fig. 3). Moreover, Pi level between GROUND-30day and HU-30day was similar (Table 1). Sample limitation prevented the assessment of other groups.

**Table 1:** Descriptive information from both the spaceflight study to the International Space Station, and the ground-based hind limb unloading (HU) study.

	Spaceflight study		Hind limb unloading study	
	GROUND	FLIGHT	GROUND-30day	HU-30day
FGF23 (pg/ml)	26.14(12.45)	27.10(14.79)	NA	NA
Pi (mg/dL)	4.26(0.29)	4.06(0.18)	7.21(3.27)	8.39(3.62)
Gastrocnemius mass (g)	159.0(13.1)	134.6(18.8)#	NA	NA

Data are presented mean(SD). # indicates diffference for FLIGHT vs GROUND at *p* = 0.0035 via unpaired t-test. NA: data not collected (Gastrocnemius mass) or presented in Fig. 3 (FGF 23).

A reduction in plasma αKlotho levels was observed in both FLIGHT and HU mice vs controls. While a mechanistic/causal link between weight-bearing conditions and αKlotho are not determined, both reduced weight-bearing conditions did result in lowered αKlotho. For HU mice, levels of αKlotho increased following exercise, with partial recovery in the running group and full recovery in the climbing group, suggesting an important relationship between exercise/potentially elevated weight-bearing and αKlotho level improvement. These data are aligned with recovery of joint health upon a return to full weight-bearing with exercise^15^. Moreover, for FLIGHT mice, where muscle masses were collected, the decrease in αKlotho post-flight occurred with reduced gastrocnemius mass, which itself is not unexpected but αKlotho may also be affected by skeletal muscle activity^18^.

When considering the importance αKlotho plays in maintaining musculoskeletal skeletal health, viewed within the context of the adverse effects of microgravity on bone, as well as muscle, these findings suggest an important link between this hormone level and skeletal health. Histologic changes of bone with altered kidney physiology in relation to these hormonal changes are needed to better understand this relationship. The similar changes in αKlotho found in both FLIGHT mice and HU mice suggest that HU mice may provide an appropriate terrestrial analog for examining the relationship between αKlotho and health along the musculoskeletal tissue-kidney axis.

## Methods

The animal and environmental details of the ISS study (Rodent Research-9 mission), the accompanying HU study, and all approved IACUC protocols from Wake Forest School of Medicine, NASA Ames, and the Kenney Space Center have been published^15^. All mice were male, C57BL/6 (Jackson Labs) that were 10 weeks at the start of each study.

### ISS mission

Groups included FLIGHT and GROUND control^15^. This investigation had access to *n* = 4–5 plasma samples/group collected after 35 days on orbit; all available samples were analyzed for this study.

### HU study

Groups included weight-bearing GROUND mice, or HU via tail suspension, as described^15^. On Day 30, plasma was isolated from a cohort of GROUND (GROUND-30day) and HU (HU-30day) mice. Remaining HU mice were then removed from tail suspension and were thus weight-bearing the remainder of the study. All mice that were previously GROUND or HU were enrolled into one of 3 exercise groups, as described^15^, from Days 31–80, performing: 1] no exercise; 2] climbing exercise 3X weekly, or; 3] running exercise 3X weekly in order to determine if recovery was possible with aerobic (running) or a resistance (climbing) regimen, yielding the following six groups: GROUND-No exercise; GROUND-Climbing; GROUND-Running; HU-No exercise; HU-Climbing; and HU-Running. Climbing exercises were performed 3X/week,. This investigation had access to *n* = 4 plasma samples/group, all of which were analyzed.

Blood was collected in [K2]ethylenediaminetetraacetic acid (EDTA)-containing syringes by cardiac puncture and centrifuged for 10 min at 3000 rpm at 4 °C; Plasma was isolated and ELISA was used to detect αKlotho (R&D Systems™ #DY5334) and FGF23 (Abcam #ab213863); a commercial Pi kit was also used (Pointe Scientific #P7516-500).

Comparisons between FLIGHT and GROUND mice were performed using a two-way unpaired t-test; ANOVA was performed for the HU study, with Tukeys post hoc tests. α ≤ 0.05; the assumption of equal variance was used to test equality of variance. Analyses were performed using GraphPad Prizm 8.4.0. The data that support the findings of this study are available on request from the corresponding author.

### Reporting summary

Further information on research design is available in the Nature Research Reporting Summary linked to this article.

## Supplementary information

Reporting Summary

## Data Availability

The authors declare that data supporting the findings of this study are available within the figures of the article, and/or are available on request from the corresponding author (J.S.W.)
