# Supplementary material for: αKlotho decreases after reduced weight-bearing from both spaceflight and hindlimb unloading
Source: NPJ Microgravity. 2022 Jun 2;8:18. doi: 10.1038/s41526-022-00203-w (PMC9163032; doi:10.1038/s41526-022-00203-w)
Supplement: Supplementary file 1 — Reporting Summary [file 41526_2022_203_MOESM1_ESM.pdf]

nature portfolio

Corresponding author(s): Jeffrey Willey

Last updated by author(s): Mar 17, 2022

Reporting Summary

Nature Portfolio wishes to improve the reproducibility of the work that we publish. This form provides structure for consistency and transparency in reporting. For further information on Nature Portfolio policies, see our Editorial Policies and the Editorial Policy Checklist.

Statistics

For all statistical analyses, confirm that the following items are present in the figure legend, table legend, main text, or Methods section.

n/a	Confirmed	
☐	☒	The exact sample size (n) for each experimental group/condition, given as a discrete number and unit of measurement
☐	☒	A statement on whether measurements were taken from distinct samples or whether the same sample was measured repeatedly
☐	☒	The statistical test(s) used AND whether they are one- or two-sided<br>*Only common tests should be described solely by name; describe more complex techniques in the Methods section.*
☒	☐	A description of all covariates tested
☐	☒	A description of any assumptions or corrections, such as tests of normality and adjustment for multiple comparisons
☐	☒	A full description of the statistical parameters including central tendency (e.g. means) or other basic estimates (e.g. regression coefficient) AND variation (e.g. standard deviation) or associated estimates of uncertainty (e.g. confidence intervals)
☐	☒	For null hypothesis testing, the test statistic (e.g. F, t, r) with confidence intervals, effect sizes, degrees of freedom and P value noted<br>*Give P values as exact values whenever suitable.*
☒	☐	For Bayesian analysis, information on the choice of priors and Markov chain Monte Carlo settings
☒	☐	For hierarchical and complex designs, identification of the appropriate level for tests and full reporting of outcomes
☒	☐	Estimates of effect sizes (e.g. Cohen's d, Pearson's r), indicating how they were calculated

Our web collection on statistics for biologists contains articles on many of the points above.

Software and code

Policy information about availability of computer code

Data collection	Excel 2016
Data analysis	GraphPad Prizm version 8.4

For manuscripts utilizing custom algorithms or software that are central to the research but not yet described in published literature, software must be made available to editors and reviewers. We strongly encourage code deposition in a community repository (e.g. GitHub). See the Nature Portfolio guidelines for submitting code & software for further information.

Data

Policy information about availability of data

All manuscripts must include a data availability statement. This statement should provide the following information, where applicable:

- Accession codes, unique identifiers, or web links for publicly available datasets
- A description of any restrictions on data availability
- For clinical datasets or third party data, please ensure that the statement adheres to our policy

Provide your data availability statement here.

Field-specific reporting

Please select the one below that is the best fit for your research. If you are not sure, read the appropriate sections before making your selection.

☒ Life sciences ☐ Behavioural & social sciences ☐ Ecological, evolutionary & environmental sciences

For a reference copy of the document with all sections, see nature.com/documents/nr-reporting-summary-flat.pdf

Life sciences study design

All studies must disclose on these points even when the disclosure is negative.

Sample size	Given the nature of the "Brief Communication" format and limited space, we cite a previous study that detailed the sample size justification (was a spaceflight study to the International Space Station) as being determined at the programmatic level by NASA. The samples for this hypothesis generating study was more limited, given that we could only test samples provided to us by a PI on that flight study.
Data exclusions	No samples that were collected were excluded, which was stated in the text
Replication	Replication was not performed due to the limited tissue samples available.
Randomization	As per sample size, the selection of groups were described in another paper that details that we had to group mice for the spaceflight study in whole cages (n=10), for if we randomized and placed the mice into new cages for launch to the space station, then we could lose some to aggression as they reestablished a hierarchy
Blinding	The technician performing the ELISAs were blinded to the plasma samples

Reporting for specific materials, systems and methods

We require information from authors about some types of materials, experimental systems and methods used in many studies. Here, indicate whether each material, system or method listed is relevant to your study. If you are not sure if a list item applies to your research, read the appropriate section before selecting a response.

Materials & experimental systems

n/a	Involved in the study	
☒	☐	Antibodies
☒	☐	Eukaryotic cell lines
☒	☐	Palaeontology and archaeology
☐	☒	Animals and other organisms
☒	☐	Human research participants
☒	☐	Clinical data
☒	☐	Dual use research of concern

Methods

n/a	Involved in the study	
☒	☐	ChIP-seq
☒	☐	Flow cytometry
☒	☐	MRI-based neuroimaging

Animals and other organisms

Policy information about studies involving animals; ARRIVE guidelines recommended for reporting animal research

Laboratory animals	We state that these were male, C57BL/6 mice (Jackson labs) that were 10 weeks at the start of each study.
Wild animals	No Wild animals
Field-collected samples	N/A
Ethics oversight	We identify that these studies were approved by the IACUCs at Wake Forest School of Medicine, NASA Ames, and the Kennedy Space Center

Note that full information on the approval of the study protocol must also be provided in the manuscript.
